# High Throughput Sequencing Identifies MicroRNAs Mediating α-Synuclein Toxicity by Targeting Neuroactive-Ligand Receptor Interaction Pathway in Early Stage of *Drosophila* Parkinson's Disease Model

**DOI:** 10.1371/journal.pone.0137432

**Published:** 2015-09-11

**Authors:** Yan Kong, Xijun Liang, Lin Liu, Dongdong Zhang, Chao Wan, Zhenji Gan, Liudi Yuan

**Affiliations:** 1 Department of Biochemistry and Molecular Biology, School of Medicine, Southeast University, Nanjing, Jiangsu Province, 210009, China; 2 MOE Key Laboratory of Model Animal for Disease Study, Model Animal Research Center, Nanjing University, Nanjing, 210061, China; 3 The Key Laboratory of Developmental Genes and Human Disease, Ministry of Education, Institute of Life Science, Southeast University, Nanjing, 210009, China; Thomas Jefferson University, UNITED STATES

## Abstract

Parkinson’s disease (PD) is a prevalent neurodegenerative disorder with pathological features including death of dopaminergic neurons in the substantia nigra and intraneuronal accumulations of Lewy bodies. As the main component of Lewy bodies, α-synuclein is implicated in PD pathogenesis by aggregation into insoluble filaments. However, the detailed mechanisms underlying α-synuclein induced neurotoxicity in PD are still elusive. MicroRNAs are ~20nt small RNA molecules that fine-tune gene expression at posttranscriptional level. A plethora of miRNAs have been found to be dysregulated in the brain and blood cells of PD patients. Nevertheless, the detailed mechanisms and their *in vivo* functions in PD still need further investigation. By using *Drosophila* PD model expressing α-synuclein A30P, we examined brain miRNA expression with high-throughput small RNA sequencing technology. We found that five miRNAs (dme-miR-133-3p, dme-miR-137-3p, dme-miR-13b-3p, dme-miR-932-5p, dme-miR-1008-5p) were upregulated in PD flies. Among them, miR-13b, miR-133, miR-137 are brain enriched and highly conserved from *Drosophila* to humans. KEGG pathway analysis using DIANA miR-Path demonstrated that neuroactive-ligand receptor interaction pathway was most likely affected by these miRNAs. Interestingly, miR-137 was predicted to regulate most of the identified targets in this pathway, including dopamine receptor (DopR, D2R), γ-aminobutyric acid (GABA) receptor (GABA-B-R1, GABA-B-R3) and N-methyl-D-aspartate (NMDA) receptor (Nmdar2). The validation experiments showed that the expression of miR-137 and its targets was negatively correlated in PD flies. Further experiments using luciferase reporter assay confirmed that miR-137 could act on specific sites in 3’ UTR region of D2R, Nmdar2 and GABA-B-R3, which downregulated significantly in PD flies. Collectively, our findings indicate that α-synuclein could induce the dysregulation of miRNAs, which target neuroactive ligand-receptor interaction pathway *in vivo*. We believe it will help us further understand the contribution of miRNAs to α-synuclein neurotoxicity and provide new insights into the pathogenesis driving PD.

## Introduction

Parkinson’s disease (PD) is the second most prevalent neurodegenerative disorder affecting the elderly population [1]. Its predominant pathological features are death of dopaminergic (DA) neurons in the substantia nigra pars compacta and intraneuronal accumulations of Lewy bodies [2]. As the main component of Lewy bodies, α-synuclein contributes to PD by aggregation into insoluble filaments. Multiplication of α-synuclein or mutations such as A53T, A30P and E46K were found in familial forms PD patients [3–5].However, the detailed mechanisms underlying α-synuclein induced neurotoxicity in PD still need further investigation.

PD animal models have been established by ectopic expression of human α-synuclein in yeast, *Caenorhabditis elegans*, *Drosophila melanogaster*, rat, mouse, and non-human primates [6–11]. *Drosophila* models have been widely used to study neurodegenerative diseases including Alzheimer’s disease (AD), Huntington’s disease (HD) and PD [8, 12, 13]. In addition to the advantages of short lifespan and convenience for genetic manipulation, *Drosophila* conceives complicated central and peripheral nervous systems which are analogous to those of human. Panneuronal expression of human wild type and mutant α-synuclein (A53T and A30P) demonstrate adult onset PD pathological features including DA neuronal loss, decreased dopamine level, impaired locomotive ability and shortened lifespan [8, 14, 15]. *Drosophila* models provide efficient tools for screening genes participate in PD and potential drugs against PD.

MicroRNAs are ~20nt small RNA molecules that fine-tune gene expression at posttranscriptional level [16]. They usually bind to 3’UTR of target mRNA and lead to translational inhibition or target degradation. It is estimated that more than half of human genes are regulated by miRNAs and the regulatory mechanisms are highly conserved among invertebrates and vertebrates. Since the discovery in 1990s, miRNAs have been found to exert essential roles in development, homeostasis and diseases. A plethora of miRNAs have been found to be dysregulated in the brain and blood of PD patients [17–20]. However, the underlying mechanisms and their functions in PD are still elusive.

In the present study, we examined the expression of miRNAs in a PD *Drosophila* model expressing α-synuclein by high throughput small RNA sequencing technology. We found that five miRNAs (dme-miR-133-3p, dme-miR-137-3p, dme-miR-13b-3p, dme-miR-932-5p, dme-miR-1008-5p) were upregulated in PD flies. Among them, miR-13b, miR-133, miR-137 are brain enriched and highly conserved from *Drosophila* to *Homo sapiens*. Validation experiment using qRT-PCR confirmed that these miRNAs were elevated in PD flies. KEGG pathway analysis indicated that neuroactive-ligand receptor interaction pathway was most likely affected by these miRNAs. Further studies showed miR-137 targeted multiple molecules in this pathway as predicted, including dopamine receptor (DopR, D2R), GABA receptor (GABA-B-R1, GABA-B-R3) and NMDA receptor (Nmdar2). The mRNA levels of these molecules were significantly decreased in PD flies. Our findings indicated that α-synuclein could induce the dysregulation of miRNAs, which target neuroactive ligand receptor interaction pathway *in vivo*.

## Materials and Methods

### Fly stocks and maintenance

The *elav*-C155 and UAS- α-synuclein flies were obtained from Bloomington Stock Center (Indiana University, USA). Flies were raised in standard yeast agar food at 25°C with a 12/12 hours light/dark cycle. After backcrossing with *w* ^118^ flies for 6 generations, *elav*-C155 virgin flies were crossed with *w* ^118^ or UAS- α-synuclein A30P males. The F1 generation offspring expressed α-synuclein in panneuronal manner and were used for further experiments.

### Lifespan analyses

Two days after the eclosion, mated males and females were discriminated and transferred to different vials. Each vial contained 10 flies and at least 100 in total for each group. The vials were changed 3 times a week and deaths were recorded. Data was presented as survival curves and analysis was performed using log-rank tests to compare between groups.

### Climbing assay

In order to characterize behavior defects in PD flies, climbing assay was performed as described previously [8]. Briefly, twenty male flies were transferred into an empty plastic vial and gently tapped to the bottom. The numbers of flies that could climb to the top (above 8cm) or remained at bottom in 18 seconds were recorded. The climbing assay was performed at least 3 times for every vial at each time point.

### High throughput sequencing for miRNAs

Total RNA of each sample (three biological repeats for PD and control fly heads) was used to prepare the miRNA sequencing library through following steps: 1) 3'-adapter ligation with T4 RNA ligase; 2) 5'-adapter ligation with T4 RNA ligase; 3) cDNA synthesis with RT primer; 4) PCR amplification; 5) extraction and purification of ~125–145 bp PCR amplified fragments (correspond to ~15–35 nt small RNAs) from the PAGE gel. After the completed libraries were quantified with Agilent 2100 Bioanalyzer, the DNA fragments in the libraries were denatured with 0.1M NaOH to generate single-stranded DNA molecules, captured on Illumina flow cells, amplified in situ and finally sequenced for 36 cycles on Illumina HiSeq2000 according to the manufacturer’s instruction. Raw sequences were generated as clean reads from Illumina HiSeq by real-time base calling and quality filtering. Subsequently, the 3’ adapter sequence was trimmed from the clean reads and the reads with lengths shorter than 15 nt were discarded. As the 5’-adaptor was also used as the sequencing primer site, the 5’-adaptor sequence is not present in the sequencing reads. The trimmed reads (length ≥ 15 nt) were aligned to the fly pre-miRNA in miRBase 21, using novoalign software. The miRNA expression levels were measured and normalized as transcripts per million of total aligned miRNA reads (TPM). When comparing profile differences two groups of samples (PD and Control), the “fold change” (i.e. the ratio of the group averages) and p-value were caculated. miRNAs having fold changes ≥ 1.2, P-value ≤0.05 or fold change ≥ 2.0 were selected as the differentially expressed miRNAs.

### qRT-PCR for miRNA

Quantitative real-time PCR (qRT-PCR) analysis was performed to validate the differently expressed mRNA in PD flies. Fist strand cDNA was sysnthesized using M-MLV reverse transcriptase (Epicentre) according to manufacture’s instructions. The sequences of RT primers are: 5’-GATTTTGCGTGTCATCCTTG-3’ (U6); 5’-GTCGTATCCAGTGCGTGTCGTGGAGTCGGCAATTGCACTGGATACGACACAGCTG-3’ (dme-miR-133-3p); 5’-GTCGTATCCAGTGCGTGTCGTGGAGTCGGCAATTGCACTGGATACGACCTACGT-3’ (dme-miR-137-3p); 5’-GTCGTATCCAGTGCGTGTCGTGGAGTCGGCAATTGCACTGGATACGACACTCGT-3’(dme-miR-13b-3p). qPCR reaction was performed with 2×SYBR Green PCR Master Mix (Arraystar) and ViiA 7 Real-time PCR System (Applied Biosystems) with the program: 95°C for 10 min to denature DNA templates, followed by 40 cycles of 95°C for 15 s, 60°C for 60s.PCR primers for miRNAs and U6 were listed in S1 Table.

### miRNA Targets Prediction and Pathway Enrichment Analysis

The target genes of differentially expressed miRNAs were predicted by miRanda-mirSVR algorithm and then subjected to GO analysis using Database for Annotation, Visualization and Integrated Discovery (DAVID) (count cutoff 10, EASE 0.01). The significantly affected GO terms (p<0.05) in biological process, cellular component and molecular function were identified.

DIANA-miRPath is an efficient tool for analyzing the combinatorial effect of microRNAs on target pathways. We uploaded the dysregulated miRNAs and predicted potential target pathways using DIANA-microT-CDS algorithm. The significantly influenced pathways (p<0.05) were identified.

### Validation of target mRNA Expression

In order to validate the expression of predicted targets for dysregulated miRNAs, qRT-PCR was performed according to previously reported methods as mentioned before. PCR primers for mRNAs were listed in S2 Table.

### Luciferase Reporter Assay

The 3’UTR fragments flanking miR-137 targeting sites of Nmdar2, D2R and GABA-B-R3 were cloned from *Drosophila* cDNA library and inserted into pGL3-promoter vectors respectively. Each of these vectors was co-transfected with Renilla plasmid pRL-TK and dme-miR-137-3p mimics (Genepharma, Shanghai) into HEK 293 cells in 12-well plates using Lipofactamine 2000 (Invitrogen). 24 hours post-transfection, luciferase activity was measured with Dual-Glo (Promega) according to the manufacturer's instructions. PCR primers for amplification were listed in S3 Table. Mutant constructs were made by site-directed mutagenesis to replace seed sequence with BglII cleavage site.

### Statistics

Log-rank tests were performed to compare lifespan between groups. For other experiments, the significance of the difference was analyzed with Student’s *t* test using GraphPad Prism software, and p <0.05 were considered statistically significant.

## Results

### Characterization of PD *Drosophila* models

**Fig 1:**
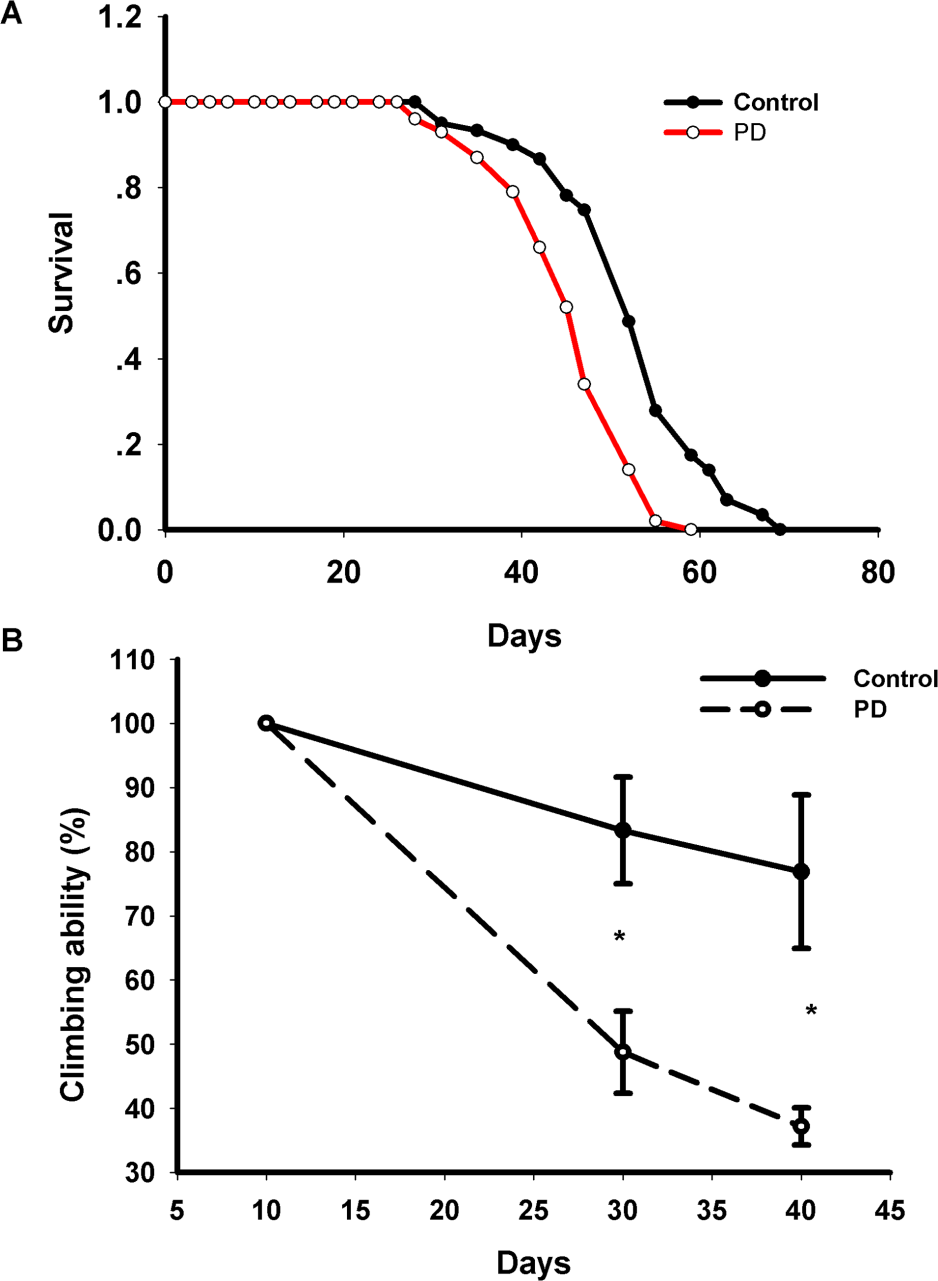
α-synuclein-induced locomotion defects and shortened lifespan. (A) Expression of A30P α-synuclein specifically in the nervous system shortened lifespan. Survival curves were compared using the log-rank test (P<0.01 between *elav*>*w* ^1118^ and *elav>*α-synuclein A30P flies). (B) There is no difference for climbing ability for flies expressing A30P α-synuclein and control genotype at day 10. In contrast, PD flies showed significant age dependent locomotive impairments at days 30 and 40 (*P<0.05). Control flies: *elav*>*w* ^1118^; PD flies: *elav>*α-synuclein (A30P).

We establised PD fly models according to literatures [8, 14, 15]. Briefly, *elav*-Gal4 flies were crossedwith UAS-α synuclein (A30P) to ectopically express human α synuclein in nervous systems. As reported previously, we found that PD flies exhibited shorter lifespan (Fig 1A) and impaired locomotive ability (Fig 1B) compared with control flies. These results indicated that *Drosophila* models successfully developed adult-onset PD like phenotype in age dependent manner. Climbing ability of PD *Drosophila* was comparable with control flies at day 10 post eclosion (Fig 1B), which was consistent with the results from Feany et al [8]. At this time point, loss of dopaminergic cells in PD *Drosophil*a was also detected. Therefore, we chose day 10 flies post eclosion as early PD stage to investigate miRNA expression profiles.

### Deep sequencing data analysis and verification

**Fig 2:**
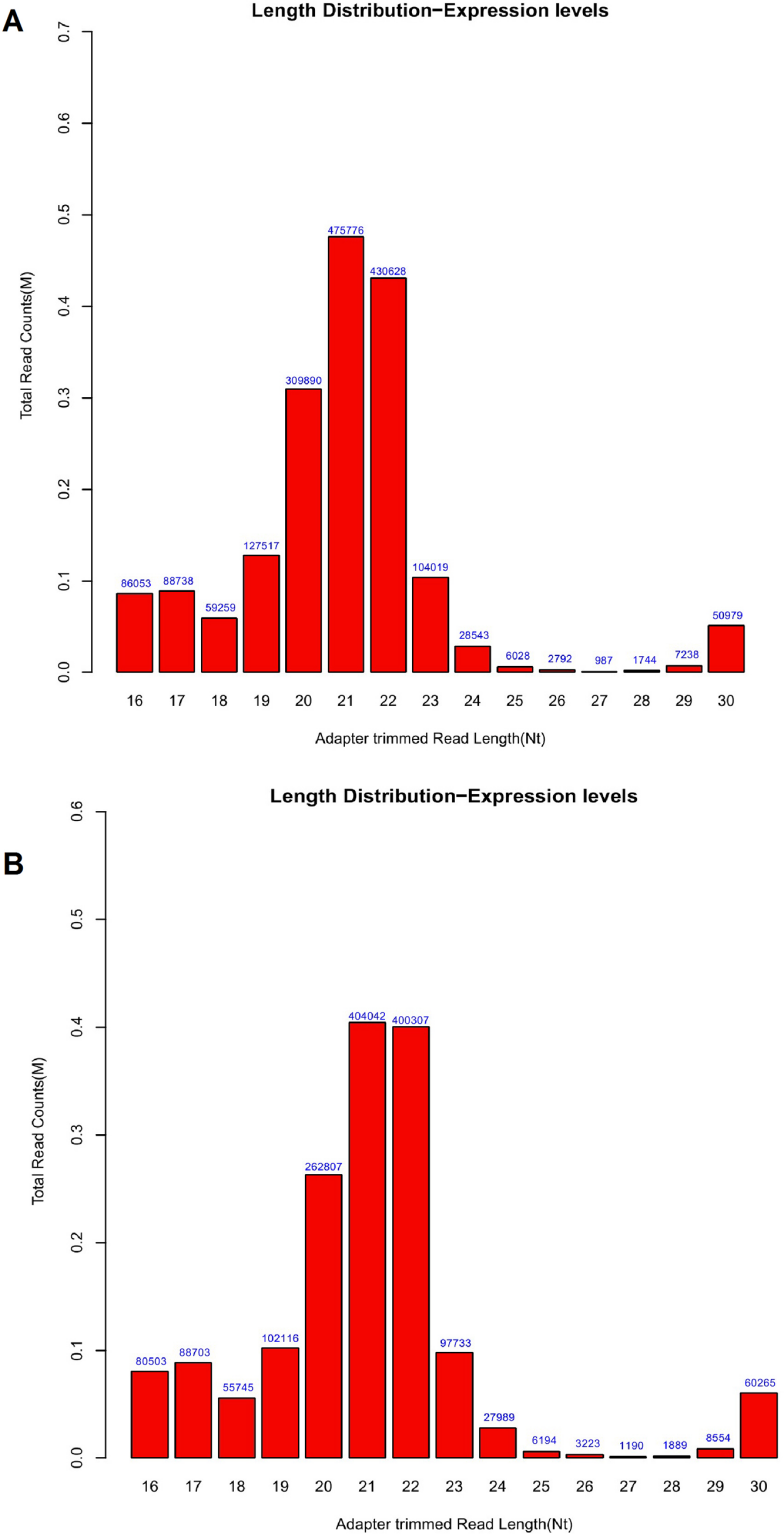
Length distribution of total small RNAs in PD and control flies. The distribution of small RNAs in control (**A)** and PD flies (**B)** was randomly selected from the data of 3 biological repeats for each group. The horizontal axis means the total read counts and vertical means the read lengths for the complete adapter-trimmed read set.

**Fig 3:**
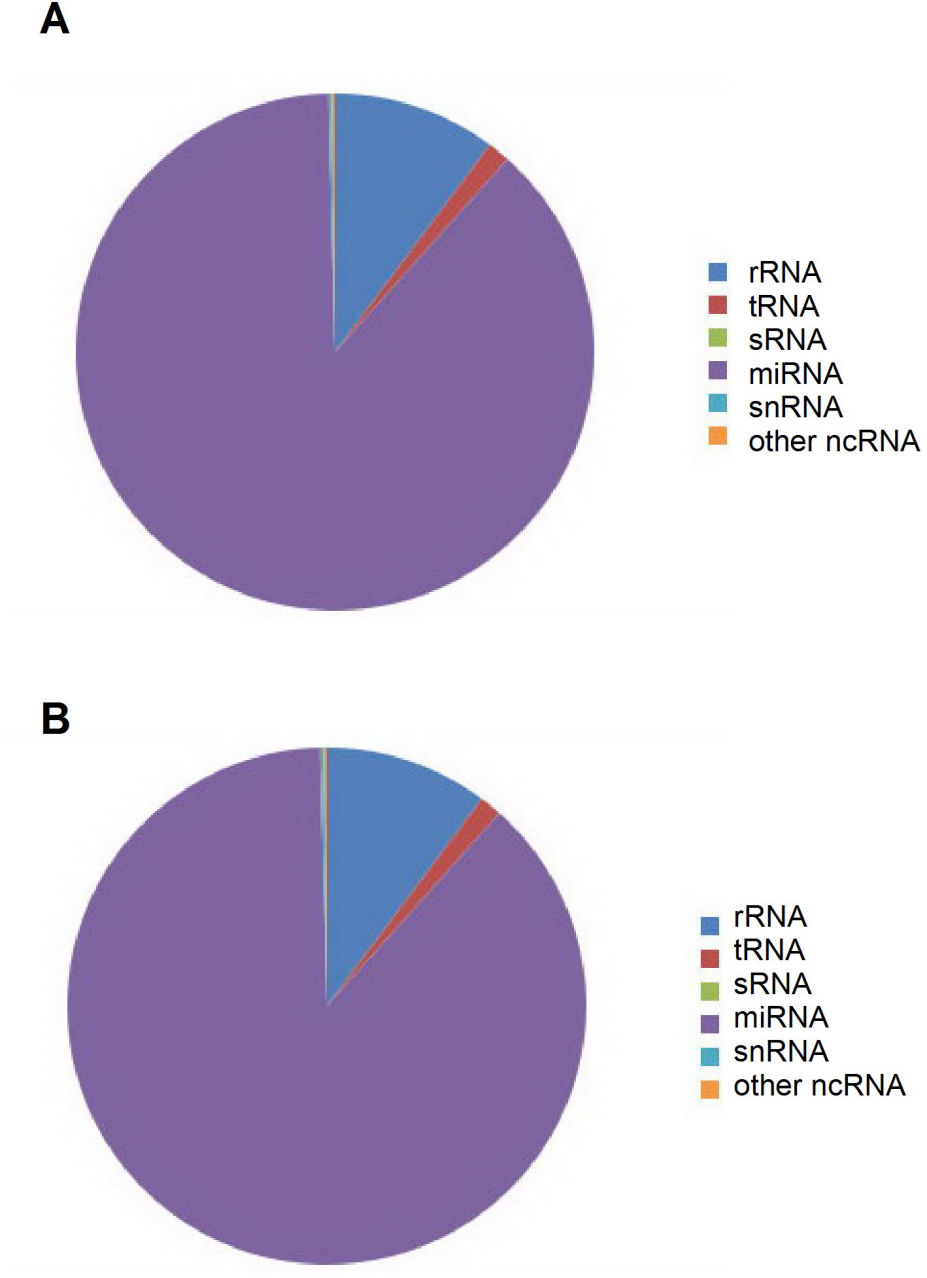
Frequency of different classes of RNA in small RNA libraries. The pie-charts represent an overview of small RNA expression in control (**A)** and PD flies (**B)**. Data were random selected from 3 biological repeats for each group. Small RNAs belonging to the miRNA constitute the majority as in control (89.5%) (top) and PD (88%) (bottom) samples.

The miRNA samples from heads of control and PD flies were sequenced using Illumina HiSeq2000 platform. The total numbers of the reads at the sequencing data processing stages are listed for each sample (3 biological repeats for control and PD flies) in Table 1. The majority of small RNAs were 20-22nt which were the typical length for miRNAs (Figs 2 and S1). The reads can be divided into several groups (miRNAs, tRNAs, rRNAs, sRNAs, snRNAs, other ncRNAs). As shown with pie charts in Figs 3 and S2, the majority was miRNAs (86.1%-90.8%).

**Table 1:** The total numbers of the reads at the sequencing data processing stages.

Sample Name	Clean Reads	Adapter-trimmed Reads (length > = 15nt)	Reads aligned to known fly pre-miRNA in miRBase 21
Control1	5,129,041	1,946,868	1,464,900
PD1	5,099,213	1,770,633	1,275,736
Control2	6,504,988	1,898,757	1,272,772
PD2	5,008,812	2,222,004	1,654,116
Control3	5,249,650	1,548,423	1,021,194
PD3	4,508,826	1,644,140	1,161,098

**Fig 4:**
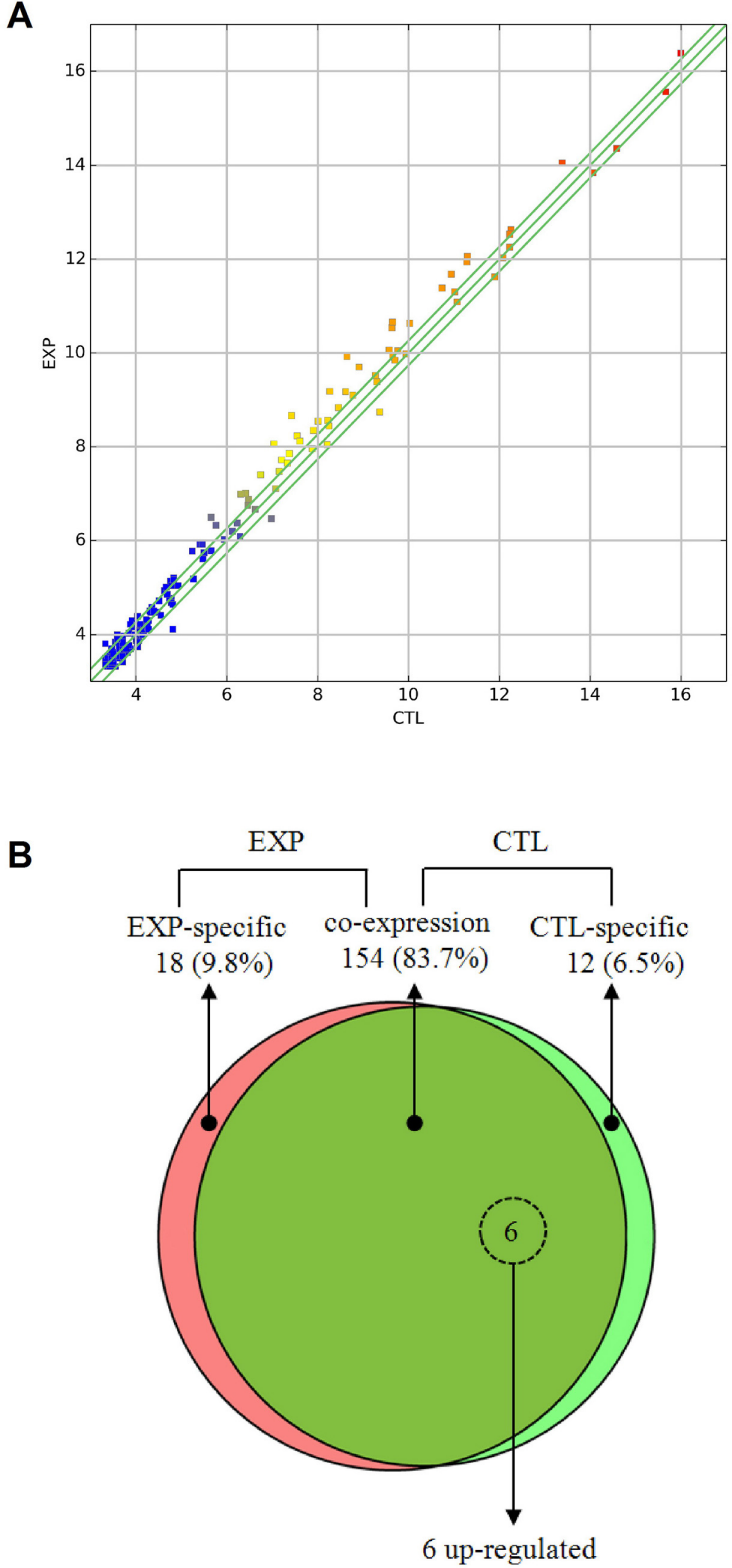
Differential expression analysis of miRNA. (A) The differentially expressed miRNAs are graphed on thescatter plot to visualize variations in miRNA expression between control and PD flies (3 biological repeats for each genotype). The values on the X-axis and Y-axis of the scatter plot are the normalized values for control and PD flies (log_2_ scaled). The green lines are fold-change lines (default fold-change value: 1.2). (B) The Venn diagram shows the distribution of 184 unique miRNAs between PD (left, red) and control flies (right, green) libraries. The overlapping section represents 154 miRNAs coexpressed in both genotypes. The dashed circles indicated 6 miRNAs that were significantly differentially expressed (dme-mir-13b-1 and dme-mir-13b-2 shares the same mature sequence dme-miR-13b-3p).

**Fig 5:**
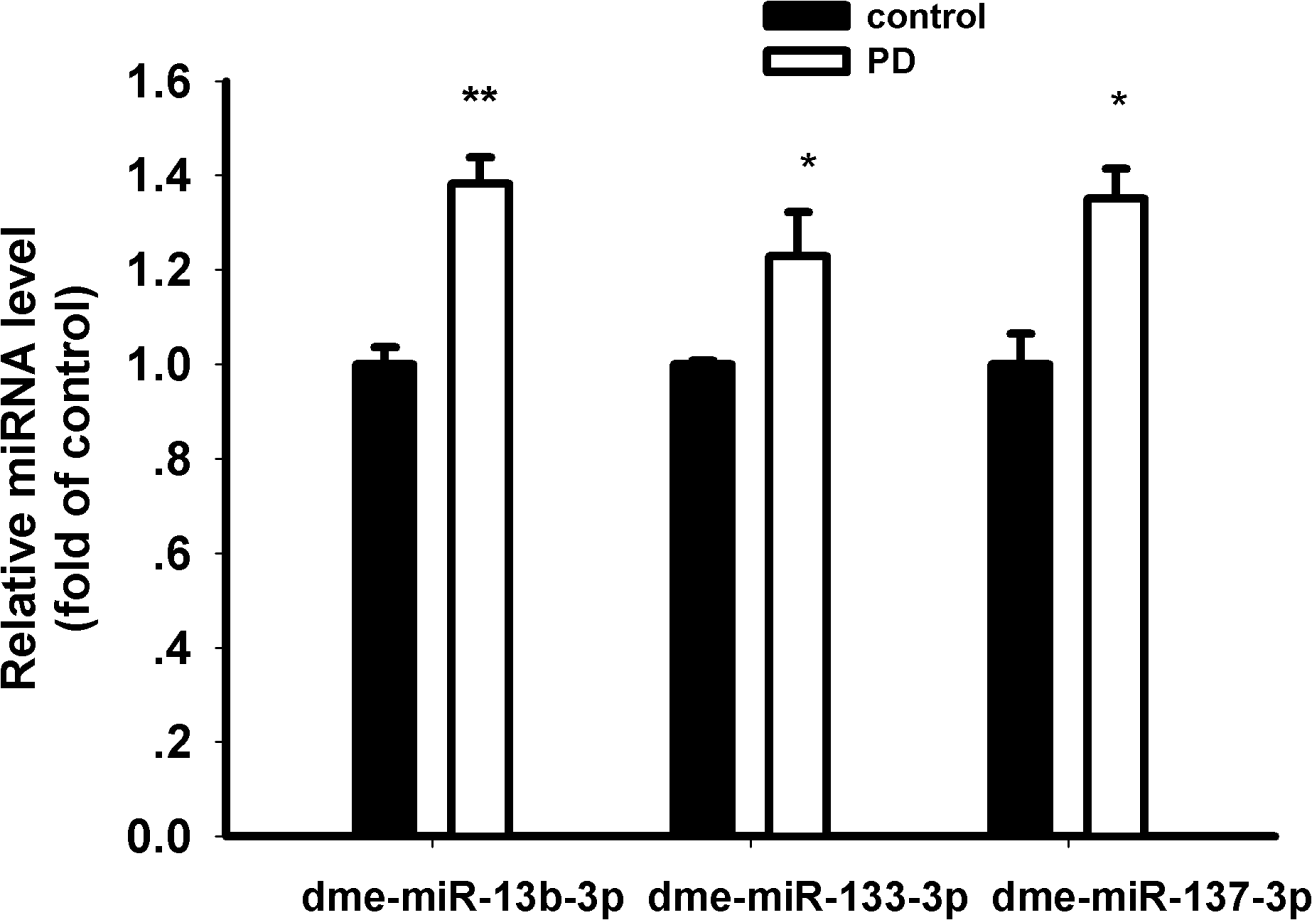
Validation analysis for dysregulated miRNA. qRT-PCR were performed to validate the expression of dme-miR-13b-3p, dme-miR-133-3p and dme-miR-137-3p in control and PD flies. The results were consistent with sequencing data. (* p<0.05, ** p<0.01).

The high-throughput sequencing results showed that 154 miRNAs (83.7% of total) were coexpressed in both control and PD flies (Fig 4). In contrast, 18 (9.8%) and 12 (6.5%) were preferentially expressed in the control or experimental groups (Fig 4B). Among 154 coexpressed miRNAs, five mature miRNAs (dme-miR-1008-5p, dme-miR-133-3p, dme-miR-137-3p, dme-miR-13b-3p, dme-miR-932-5p) were differentially expressed between PD and control groups (p<0.05) (Table 2 and S5 Table). Interestingly, all these miRNAs were upregulated in PD flies. Among them, dme-miR-133-3p, dme-miR-137-3p and dme-miR-13b-3p (the mature sequence both for dme-mir-13b-1 and dme-mir-13b-2) were highly conserved from flies to humans and enriched in nervous system. We choose them for validation using qRT-PCR. The results were consistent with miRNA sequencing data (Fig 5).

**Table 2:** Differentially expressed miRNAs.

**MATURE-ID**	**PRE-ID**	**PRE-ACC**	**MATURE-LENGTH**	**MATURE-SEQ**	**EXP vs CTL Fold change**	**EXP vs CTL P-value**
dme-miR-932-5p	dme-mir-932	MI0005820	22	UCAAUUCCGUAGUGCAUUGCAG	1.285714286	0.019803941
dme-miR-13b-3p	dme-mir-13b-2	MI0000135	22	UAUCACAGCCAUUUUGACGAGU	1.49382716	0.008027299
dme-miR-13b-3p	dme-mir-13b-1	MI0000134	22	UAUCACAGCCAUUUUGACGAGU	1.49382716	0.008027299
dme-miR-137-3p	dme-mir-137	MI0005849	22	UAUUGCUUGAGAAUACACGUAG	1.255868545	0.034111438
dme-miR-133-3p	dme-mir-133	MI0000362	22	UUGGUCCCCUUCAACCAGCUGU	1.301026694	0.009145923
dme-miR-1008-5p	dme-mir-1008	MI0005869	21	GUAAAUAUCUAAAGUUGAACU	1.228571429	0.015268072

### Functional annotations for targets of differentially expressed miRNAs

**Fig 6:**
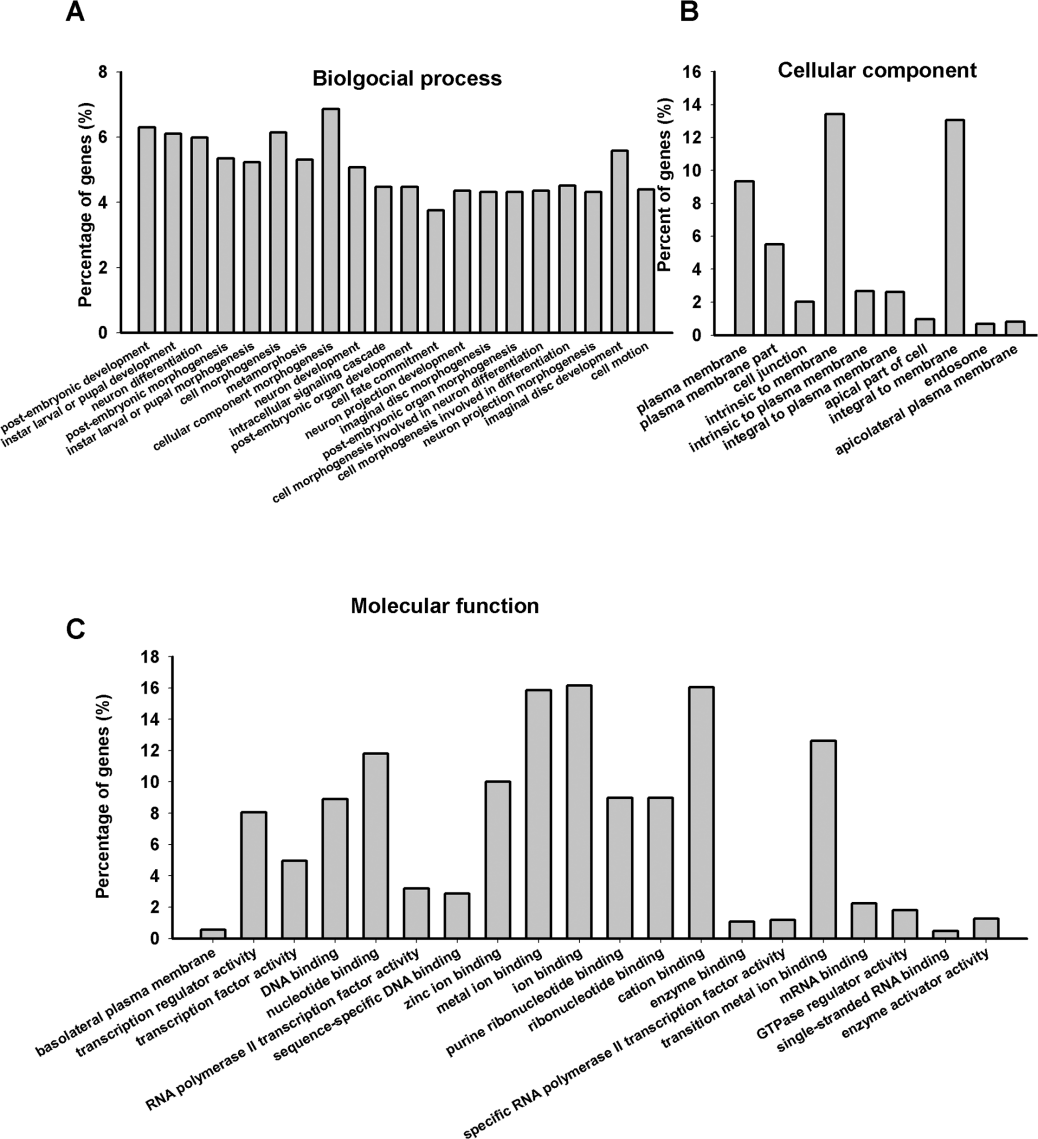
GO annotation of predicted targets for differentially expressed miRNAs. Functional annotations were performed using DAVID (count cutoff 10, EASE 0.01) to analyze predicted targets for differentially expressed miRNAs. The top 20 clusters in biological process and molecular function as well as top 10 terms in cellular component were shown (p<0.05).

As four of the dysregulated miRNAs in PD flies including dme-miR-133-3p, dme-miR-137-3p, dme-miR-13b-3p and dme-miR-932-5p were brain enriched, we predicted targets of them and then submit to DAVID for Gene Ontology analysis (Fig 6 and S7 Table). GO enrichment analysis revealed that the target genes were functionally enriched in neuron related biological process (neurodifferentiation, neuron development, neuron projection development, neuron projection morphogenesis). In addition, cell component analysis showed that these targets were enriched in the membrane proteins.

**Fig 7:**
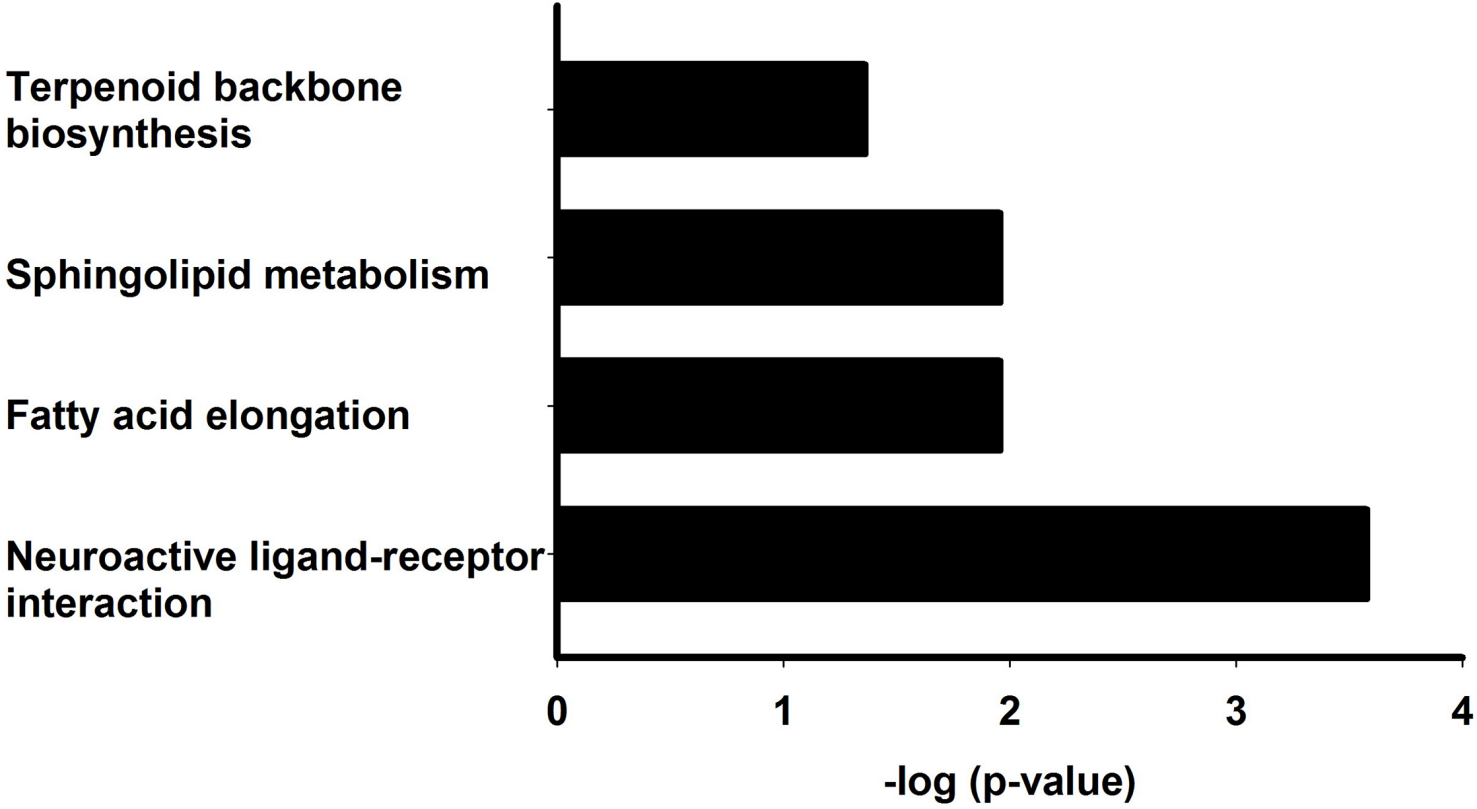
Pathway enrichment of predicted miRNA targets. DIANA miRPath v.2.0 was used for pathway functional annotation. Significant affected pathways (p<0.05) were shown. The results were displayed as–log p values.

DIANA miRPath is a powerful tool to analyze the combinational effects of miRNA on signaling pathways [21]. Using this method, we found dysregulated miRNAs significantly affect four pathways, of which neuroacitve-ligand receptor interaction was most significant (Fig 7 and Table 3). Four of the total dysregulated miRNA could target 8 genes of this pathway. As shown in S3 Fig and Table 4, miR-137-3p potentially targeted Nmdar2 (receptor for N-Acetylaspartyl glutamate and Glutamate, L-asparate, L-cysteic acid, L-homocysteic acid), GABA-B-R1/GABA-B-R3 (GABA receptor) and D2R/DopR (Dopamine receptor). Lgr3 (Relaxin receptor) and AR2 (Galanin receptor) were predicted to be targeted by miR133-3p and miR-13b-3p respectively. In addition, miR-932-5p was proposed to act on AlstR (Galanin receptor) and GABA-B-R1 (GABA receptor). These results indicated that dysregulation of miRNAs potentially lead to interruption of neuroactive-ligand receptor signaling pathway and contributed to α-synuclein induced PD flies.

**Table 3:** KEGG pathway analysis for differential expressed miRNAs.

KEGG pathway	p-value	genes	miRNAs
Neuroactive ligand-receptor interaction	0.000265508	8	4
Fatty acid elongation	0.01106502	1	1
Sphingolipid metabolism	0.01106502	3	2
Terpenoid backbone biosynthesis	0.04363707	2	2

**Table 4:** Target genes for differential expressed miRNAs in neuroactive-ligand receptor interaction pathway.

miRNA names	Targets	
dme-miR-137-3p	Nmdar2	FBgn0053513
	GABA-B-R3	FBgn0031275
	D2R	FBgn0053517
	DopR	FBgn0011582
	GABA-B-R1	FBgn0260446
dme-miR-133-3p	AR-2	FBgn0039595
dme-miR-13b-3p	Lgr3	FBgn0039354
dme-miR-932-5p	AlstR	FBgn0028961
	GABA-B-R1	FBgn0260446

### The mRNA levels of predicted targets were downregulated in PD flies

**Fig 8:**
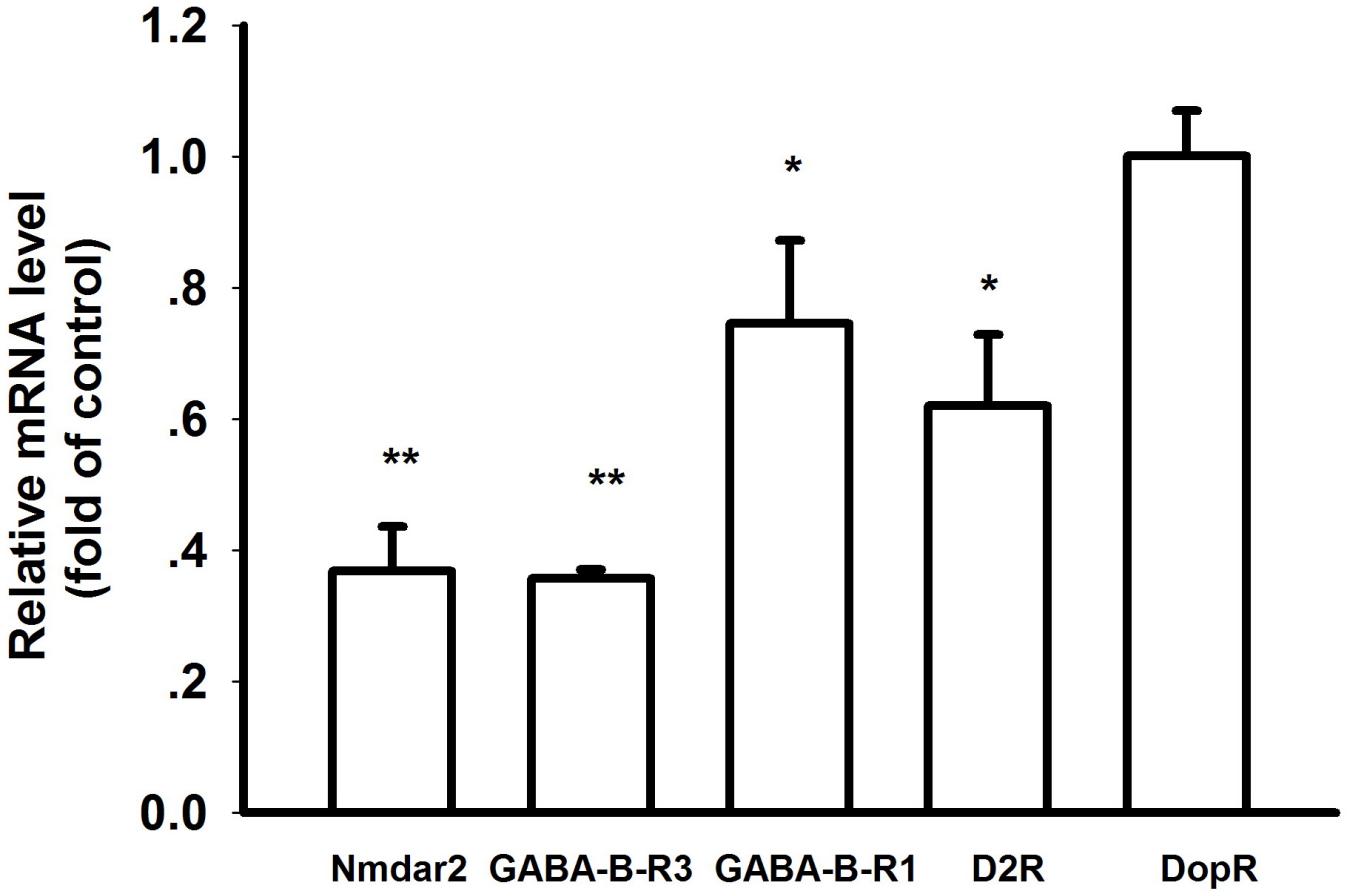
Validation analysis for targets in neuroactive ligand-receptor interaction pathway. The mRNA levels for targets were validated using qRT-PCR in control and PD flies. The results showed that the targets were significantly inhibited in PD flies. (* p<0.05, ** p<0.01).

We examined the transcriptional levels of miR-137 targets in neuroactive ligand-receptor interaction pathway. Within five predicted targets, Nmdar2, GABA-B-R3, GABA-B-R1 and D2R were confirmed to be downregulated in PD flies (Fig 8). Particularly, the NMDA receptor Nmdar2 and GABA receptor GABA-B-R3 were most significant. Interestingly, hsa-miR-137-3p was also predicted to target KEGG pathways including Glutamatergic synapse (hsa04724) (p = 0.001749507) and GABAergic synapse (hsa04727) (p = 0.007160067) by DIANA miRPath analysis. GABA-B receptor (GABRA1, GABRA6, GABBR2) and NMDA receptor (GRIN2A) were identified as hsa-miR-137-3p targets (Table 5). Our results were consistent with previous reports that PD was associated with neuroactive ligand-receptor interaction pathway [22] and miR-137 could regulate synaptogenesis and neuronal transmission [23]. The regulatory effects of miR-137 on GRIN2A expression have been confirmed in human neuronal like SH-SY5Y cells [23]. Luciferase reporter assay showed that miR-137 could target GRIN2A directly in Rats [24]. The regulatory mechanisms seemed to be highly conserved from *Drosophila* to humans.

**Table 5:** Target genes for hsa-miR-137-3p in GABAergic synapse and Glutamatergic synapse pathway in *Homo sapiens*.

	GABAergic synapse	Glutamatergic synapse
hsa-miR-137-3p targets	GABRA1	ADCY1
	PLCL1	ADCY2
	ADCY1	GRM5
	ADCY2	SLC17A6
	GABRG2	CPD
	GABBR2	PLCB1
	GAD2	PPP3CB
	GABRA6	DLGAP1
	SRC	GRM7
	CACNA1D	CACNA1D
	ADCY9	GRIN2A
	SLC6A1	ADCY9
		KCNJ3
		SLC1A2

**Fig 9:**
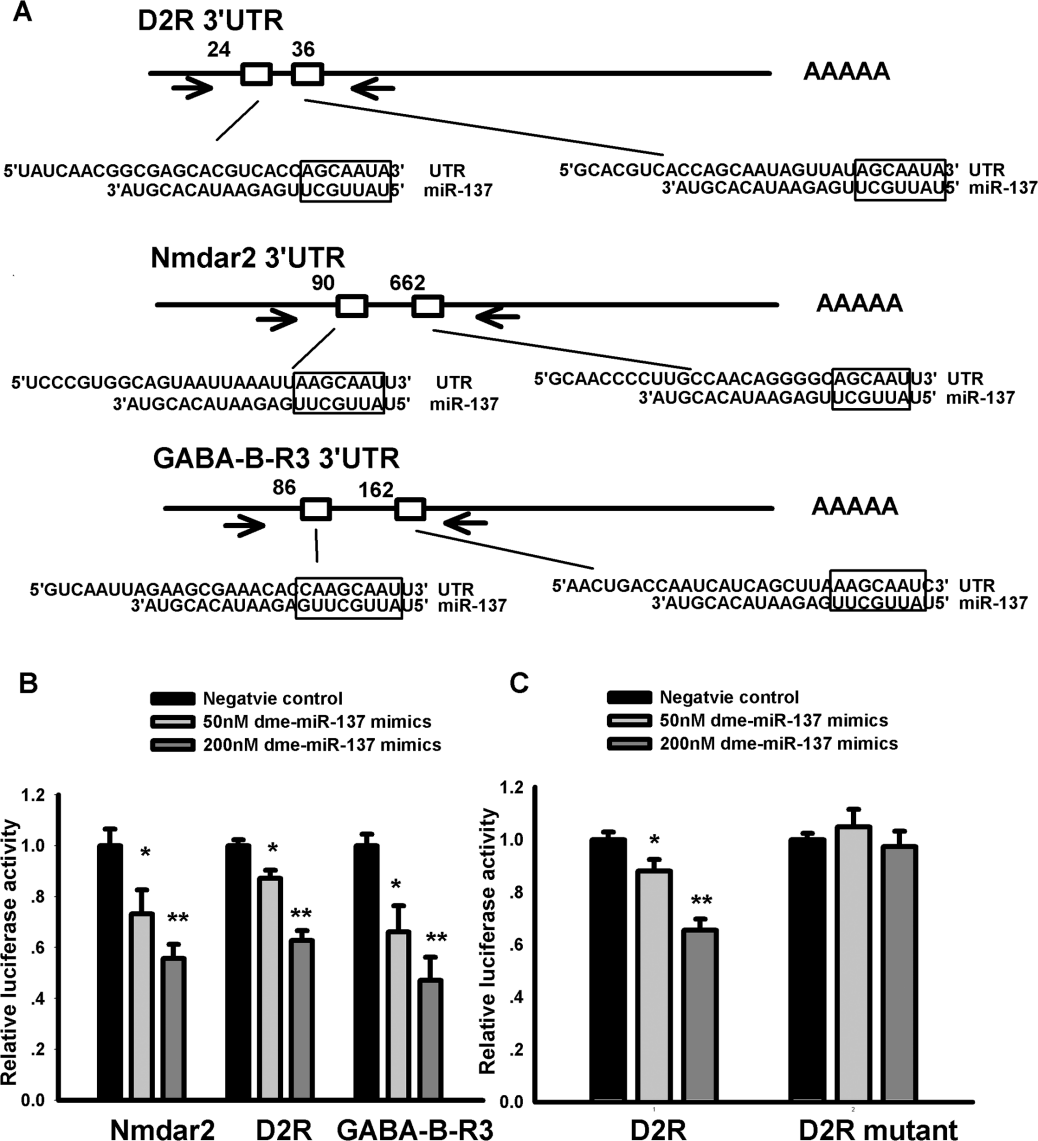
Luciferase reporter assays confirmed dme-miR-137 could inhibit the targets in neuroactive-ligand receptor interaction pathway. (A) 3′UTRs of Nmdar2, D2R and GABA-B-R3 containing dme-miR-137-3p binding sites predicted by DIANA—microT (shown in square) were cloned into pGL3-promoter vectors. Arrows indicated the location of primers used for amplification. (B) The pGL3-promoter vector carrying Nmdar2, D2R and GABA-B-R3 3’UTR fragments flanking miR-137 targeting sites were co-transfected with Renilla plasmid pRL-TK as well as dme-miR-137-3p mimics into HEK 293 cells. The results showed that dme-miR-137-3p could inhibit the luciferase activities for all these vectors does dependently as compared with miR-negative control. (C) The inhibitory effects were abolished when all the miR-137 targeting sites within the amplified sequences in D2R 3’UTR were mutated. (* p<0.05, ** p<0.01).

In order to further confirm dme-miR-137 could directly regulate targets in neuroactive-ligand receptor interaction pathway, we constructed luciferase reporter plasmids carrying Nmdar2, D2R and GABA-B-R3 3’UTR fragments containing miR-137 binding sites (Fig 9A). Luciferase reporter assays showed that dme-miR-137-3p could does-dependently inhibit the luciferase activities for all these vectors as compared with miR-negative control, indicating that dme-miR-137-3p could target these predicted sites (Fig 9B). Further more, when we mutated both of miR-137 binding sites in D2R 3’UTR, the inhibitory effects were abolished (Fig 9C). Taken together, these results indicate that NMDAR2, D2R and GABA-B-R3 are direct targets for dme-miR-137-3p.

## Discussion

As regulatory molecules that fine-tune gene expression at posttrascriptional level, miRNAs have been estimated to exert important roles in PD. However, their detailed *in vivo* functions are still elusive. *Drosophila* models provide powerful tools to investigate etiology and intervention methods for PD. Using high throughput small RNA sequenceing technology, we measured miRNA expression profiles of early stage PD flies and identified five dysregulated mature miRNAs (miR-13b, dme-miR-133, dme-miR-137, miR-932 and miR-1008). KEGG functional annotation analysis showed that neuroactive-ligand receptors to be potentially affected by these miRNAs, which were confirmed by qRT-PCR analysis and luciferase reporter assay. Our study proposed miRNAs as potential biomarker for early stage PD and their dysregulaton subsequently participates in PD pathogenesis by interruption of neuroactive-ligand receptor interaction pathway.

PD is a neurodegenerative disorder characterized by intraneuronal accumulation of filamentous inclusions known as Lewy body in substantia nigra. Feany and Bender established PD fly models by panneuronal expression normal and mutant forms of human α-synuclein, the main components accumulated in Lewy body [8, 14, 15]. PD flies shows intraneuronal inclusions, loss of dopamine neurons and impaired locomotive ability. It provides powerful tool to investigate underlying mechanisms for PD. Endonuclease G (EndoG) and sirtuin2 (SIRT2) have been identified contributing to α synuclein toxicity while vacuolar protein sorting 35 (VPS35), glucose phosphate isomerase 1 (GPI), tumor necrosis factor receptor-associated protein 1 (TRAP1), nuclear factor erythroid 2-related factor 2 (Nrf2), Rab1a, Rab8, histone deacetylase 6 (HDAC6), PTEN induced putative kinase 1 (Pink1), Cu/Zn superoxid Dismutase (SOD), methionine sulfoxide reductase A (MSRA), parkin as well as heat shock cognate 70-kd protein (Hsp70) could intervene PD progression [25–37]. Drugs currently used to treat this disorder have been tested in this model. Dopamine agonist (such as L-DOPA, pergolide, bromocriptine, and 2,3,4,5-tetrahydro-7,8-dihydroxy-1-phenyl-1H-3-benzazepine) and prototypical muscarinic cholinergic receptor antagonist are found to be effective to restore climbing defects, confirming the utility of this model in screening PD drugs [38]. Since then, a number of potential drugs have been clarified. Spermidine, GABA, L-ascorbic acid, nordihydroguaiaretic acid, fendiline, geldanamycin, isorhynchophylline (IsoRhy), curcumin, epicatechin gallate, mannitol, sodium butyrate, S-methyl-L-cysteine (SMLC) as well as plant extracts including cinnamon extract precipitation (CEppt), *Ocimum sanctum* leaf extract extract, *E*. *citriodora* extract and Regrapex-R have been proved to ameliorate PD pathogenesis [35, 39–52]. Taken together, these results demonstrate that α synuclein induced PD fly models provide efficient tool for clarifying etiology and screening potential drugs for this disorder.

MiRNAs regulate gene expression at posttranscriptional level, which plays important roles in neurodegenerative diseases. Expression profiling analysis has identified a variety of miRNAs dysregulated in brain regions and blood samples from PD patients and animal models [17–20]. As α-synuclein inclusions is the major component of Lewy body, miRNAs (miR-34b, miR-34c, miR-153 and miR-7) could target 3’UTR of α-synuclein and ameliorate its toxic effects [53, 54]. In addition, miRNAs could also act on downstream signaling molecules mediating α-synuclein toxicity. Midbrain dopamine neuron (DA) specific miR-133b was found to target paired-like homeodomain transcription factor (Pitx3) and regulate DA neurons differentiation and activity [55]. MiR-128 could repression of transcription factor EB (TFEB) in both A9 and A10 DA neurons which further inhibits mTOR activation and defense against α-synuclein toxicity [56]. However, these findings were obtained from *in vitro* studies. Further experiments using genetic modified animal models are required to clarify detailed miRNA functions in PD. With advantages discussed previously, *Drosophila* PD models could contribute to elucidation PD related miRNA functions *in vivo*.

Our study using high throughput sequencing of miRNAs identified miR-13b, miR-133, miR-137, miR-932 and miR-1008 consistently upregulated in early stage PD flies. Among the dysregulated miRNAs, miR-13b, miR-133 and miR-137 were highly conserved from *Drosophila* to *H*. *sapiens* and their expression was validated by qRT-PCR. MiR-13b’s human homologue is miR-499 [57] that expressed in brain region and its polymorphism is associated with ischemic stroke [58]. Previously, we found miR-13b was also upregulated in adult onset AD flies [59]. These results indicate that miR-13b/miR-499 play important roles in pathogenesis of brain insults. MiR-133a and miR-133b are human orthologs of dme-miR-133 and enriched in human brain. Exosomes containing miR-133b from mesenchymal stem cells (MSCs) regulate neurite outgrowth of neural cells [60]. Morphine regulates dopaminergic neuron differentiation via miR-133b [61]. In addition to its physical functions, miR-133b is essential for functional recovery after spinal cord injury in adult zebrafish [62]. By targeting Pitx3, miR-133b was found to regulate the maturation and function of midbrain dopaminergic neurons, contributing to PD pathogenesis [55]. MiR-137 is also a highly conserved miRNA and exerts important roles in neuronal development and diseases. By regulating expression of nuclear receptor tailless (TLX) and lysine-specific demethylase 1 (LSD1) in neural stem cells, miR-137 controls the dynamics between neural stem cell proliferation and differentiation during neural development [63]. MiR-137 could also regulate neuronal maturation by targeting ubiquitin ligase mind bomb-1 [64]. Recently, it was reported that miR-137 and its seed-similar fly homologue miR-1000 regulated vesicular glutamate transporter (VGlut) expression and fine-tune excitatory synaptic transmission [65]. In addition, miR-137 also plays important roles in brain disorders. MiR-137 is associated with intellectual disability [66]. miR-137 is also proved to be associated with schizophrenia susceptibility, which usually accompanied with PD [67–69]. The mechanistic studies reveal that miR-137 regulates gene sets involved in synaptogenesis and neuronal transmission as well as glucocorticoid receptor-dependent signalling network, contributing to etiology of schizophrenia [23, 70]. In another neurodegenerative disorder Alzheimer’s disease, miR-137 is found to be associated with serine palmitoyltransferase (SPT) and amyloid β (Aβ) levels [71].

The reason for α-synuclein induced miRNA dysregulation *in vitro* could be explained in various mechanisms. Firstly, α-synuclein overexpression and aggregation in neuronal cells may influence signaling pathways and transcription factors that mediate miRNA expression. α-synuclein expression could influence signaling pathways including IRS-1/insulin/Akt, mTOR/S6K, MAPK, p53, GSK3β, PKC, synaptic transmission, ubiquitin protein pathway (UPS) and the autophagy pathway [72–79]. These pathways could further stimulate transcription factors and lead to miRNA dysregulation. ɑ synuclein could increase the activity of transcription factors including NRF2, NFAT, MEF2C-PGC1α, CREB, NF-κB, p53, Nurr1, and FOXP1 [74, 80–86]. We analyzed the promoter region of dme-miR-137 (5kb upstream of pre-dme-miR-137) using AliBaba2.1 based on TRANSFAC 4.0 and found three CREB binding sites as well as six NF-κB binding sites. In addition, CREB and NF-κB were also predicted to bind to hsa-miR-137 promoter, indicating the regulatory mechanisms were highly conserved. Taken together, ɑ synuclein may induce miR-137 expression by transcription factor CREB and NF-κB. Second, α-synuclein overexpression and aggregation in neuronal cells may stimulate cells to release different factors that induce miRNA expression. These factors include brain-derived neurotrophic factor, glial cell line-derived neurotrophic factor, reactive oxygen species, nitric oxide and other factors [87–90]. These factors may act on other cells and actviate relevant signaling pathways as well as downstream transcription factors and induce miRNA expression. Third, the extracellular α-synuclein aggregates may act on neurons to regulate miRNA expression. It was reported that exogenous α-synuclein fibrils induce could activate singlaing pathways including PI3/Akt, calpain-dependent CDK5, LKB1/AMPK/Raptor, leading to synaptic dysfunction and neuron death [91–94]. Extracellular alpha-synuclein may also induce miRNA expression *in vitro*. Detailed experiments are required to clarify this problem

In order to elucidate which signaling pathways potentially affected by these dysregulated miRNAs in PD flies, DIANA-miRPath analysis was performed and Kyoto Encyclopedia of Genes and Genomes (KEGG) pathway neuroactive-ligand receptor interaction was identified. Consistent with our findings, Huang et al. reported that when applying to a genome-wide association study (GWAS) dataset for Parkinson disease, extended Bayesian lasso (EBLasso) identified three significant pathways including the neuroactive-ligand receptor interaction, the primary bile acid biosynthesis pathway, and the mitogen-activated protein kinase (MAPK) signaling pathway [95]. Our validation experiments showed that downregulations of NMDA receptor (Nmdar2) and GABA receptor (GABA-B-R3) were most significant. NMDA receptor GRIN2A was also predicted to be targeted by miR-137 in *Homo sapiens*, which have been validated in human SY-SH5Y cells [23]. Luciferase reporter assay showed that miR-137 could target GRIN2A directly in Rats [24], suggesting the regulatory mechanisms seemed to be highly conserved from *Drosophila* to humans. Interestingly, Genome-wide gene-environment study identifies glutamate receptor gene GRIN2A as a Parkinson's disease modifier gene via interaction with coffee [95]. Activation of GABAB receptors within the substantia nigra pars reticulata (SNr), but not the globus pallidus (GP), reverses reserpine-induced akinesia in rats. The success of intracerebroventricular injection of baclofen suggests a potential for GABAB receptor agonists in the treatment Parkinson's disease [96]. Hillman R et al reported that GABA rescue the loss of climbing activity in this PD fly models [40]. More specifically, GABA(B) agonists baclofen and the allosteric agonists CG 7930 and GS 39783 could also ameliorate locomotive defects, which diminished when flies are cofed with the GABA(B) receptor antagonist 2-hydroxysaclofen. In contrast, GABA(A) receptor agonist muscimol has no effect. This result indicated the important roles for neuroactive-ligand pathways in PD. Next step, we will use genetic manipulations and pharmacological methods to clarify the role miRNA-targets axis we identified within this pathway in PD.

## Conclusions

Our findings indicated that α-synuclein could induce the dysregulation of highly conserved and brain enriched miRNAs, which target neuroactive ligand-receptor interaction pathway *in vivo*. We believe it will contribute to understanding miRNA functions in mediating α-synuclein toxicity and provide new insights into the pathogenesis driving PD.

## Supporting Information

S1 Fig Length distribution of total small RNAs in PD (PD2 and PD3) and control (control2 and control 3) flies. (TIF)

S2 Fig Frequency of different classes of RNA in small RNA libraries in PD (PD2 and PD3) and control (control2 and control3) flies. (TIF)

S3 Fig Predicted targets for dysreuglated *Drosophila* miRNAs in KEGG neuroactive ligand-receptor interaction pathway. The targets predicted by DIANA miRPath v.2.0 in neuroactive ligand-receptor interaction pathway were shown in red square.
(TIF)

S4 Fig Predicted targets in KEGG Glutamatergic synapse in *H*. *sapiens*. The hsa-miR-137-3p targets predicted by DIANA miRPath v.2.0 in Glutamatergic synapse pathway were shown in red square. NMDA receptor GRIN2A was identifies as potential target.
(TIF)

S5 Fig Predicted targets in KEGG GABAergic synapse in *H*. *sapiens*. The hsa-miR-137-3p targets predicted by DIANA miRPath v.2.0 in GABAergic synapse pathway were shown in red square. GABA receptors including GABRA1, GABRA6 and GABBR2 were identifies as potential targets.
(TIF)

S1 Table PCR primers for miRNAs and U6. (XLS)

S2 Table PCR primers for mRNA targets. (XLS)

S3 Table PCR primers for luciferase reporter assay. (XLS)

S4 Table Expression profile of miRNAs in control and PD flies. (XLS)

S5 Table Differentially expressed miRNAs between control and PD flies. (XLS)

S6 Table Novel miRNAs were predicted with miRDeep2. (XLS)

S7 Table GO functional enrichment annotations for the miRNA targets. (XLSX)
