# Supplementary figures and images for: High Throughput Sequencing Identifies MicroRNAs Mediating α-Synuclein Toxicity by Targeting Neuroactive-Ligand Receptor Interaction Pathway in Early Stage of Drosophila Parkinson's Disease Model
Source: PLoS One. 2015 Sep 11;10(9):e0137432. doi: 10.1371/journal.pone.0137432 (PMC4567341; doi:10.1371/journal.pone.0137432)

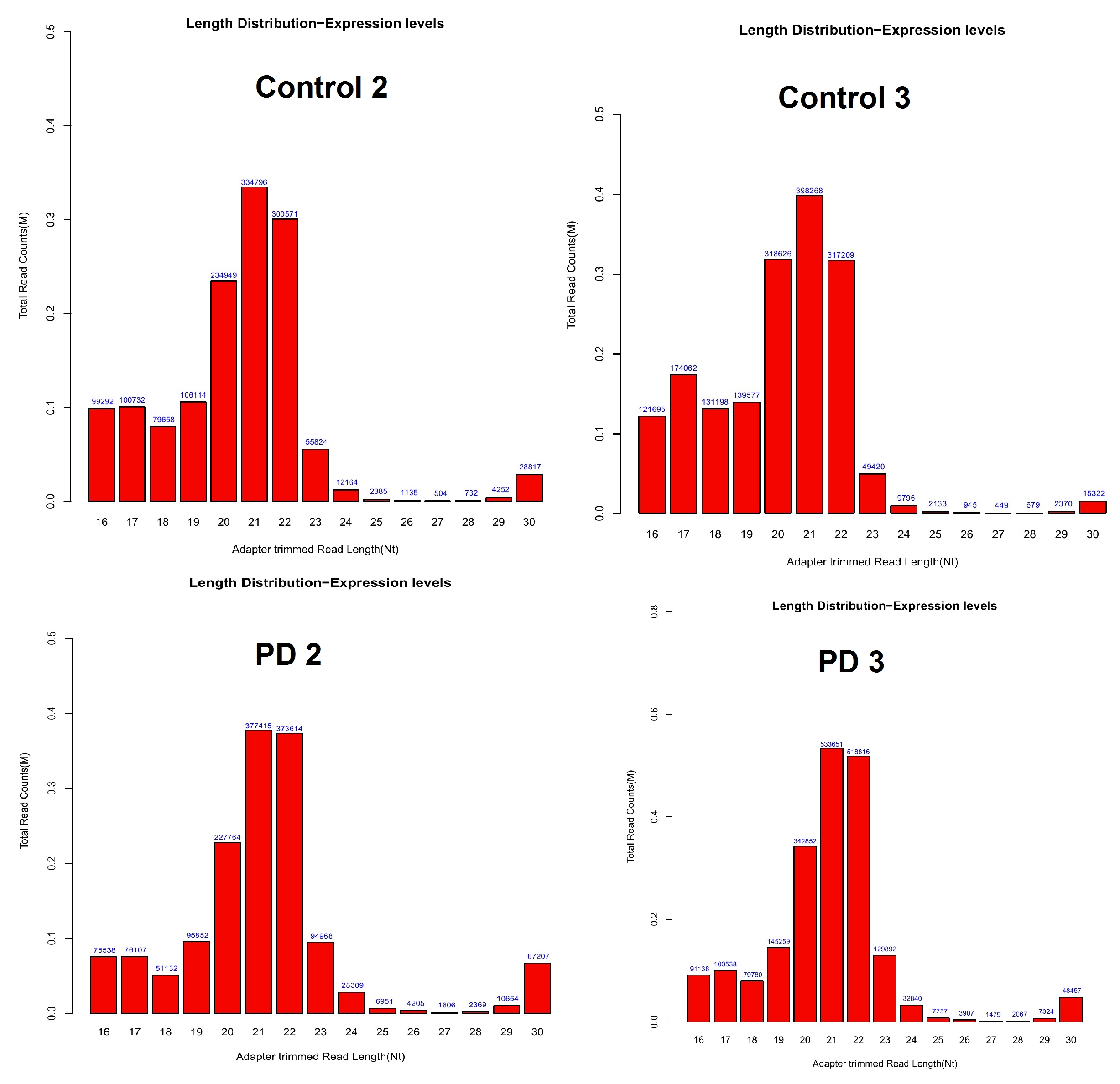

Supplement: S1 Fig — (TIF) [file pone.0137432.s001.tif]

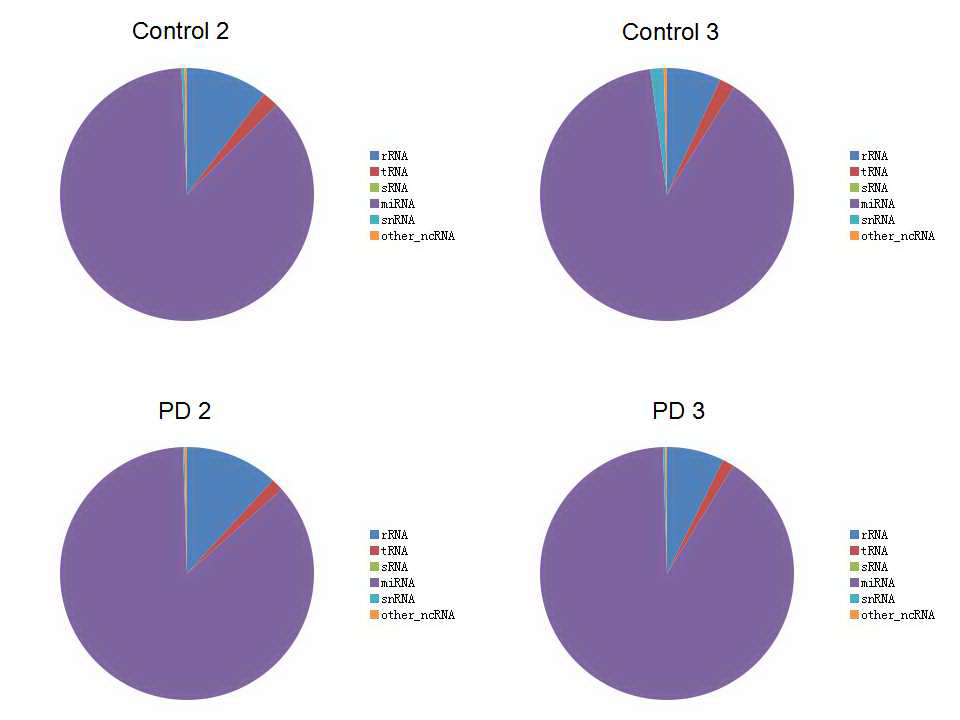

Supplement: S2 Fig — (TIF) [file pone.0137432.s002.tif]

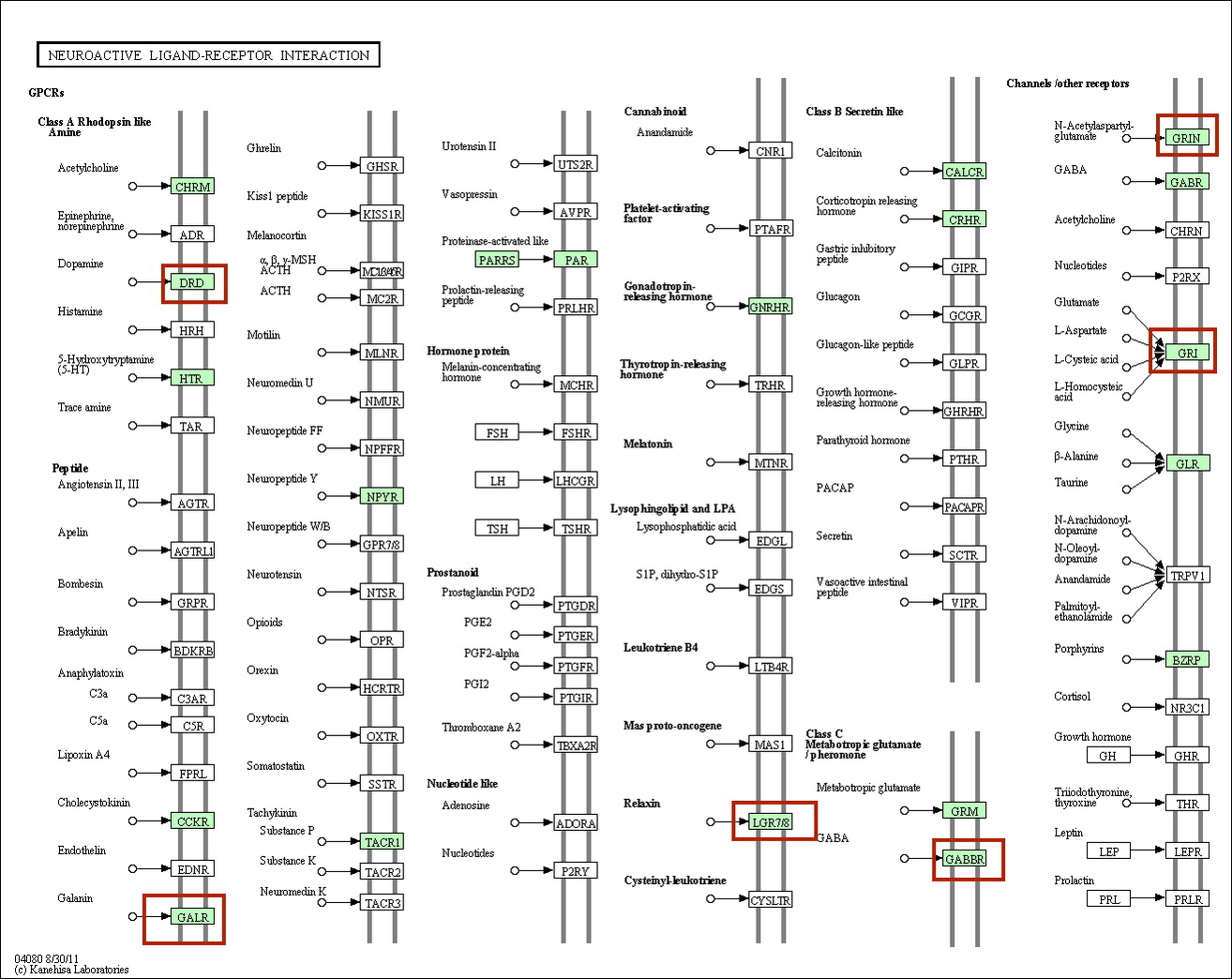

Supplement: S3 Fig — The targets predicted by DIANA miRPath v.2.0 in neuroactive ligand-receptor interaction pathway were shown in red square. (TIF) [file pone.0137432.s003.tif]

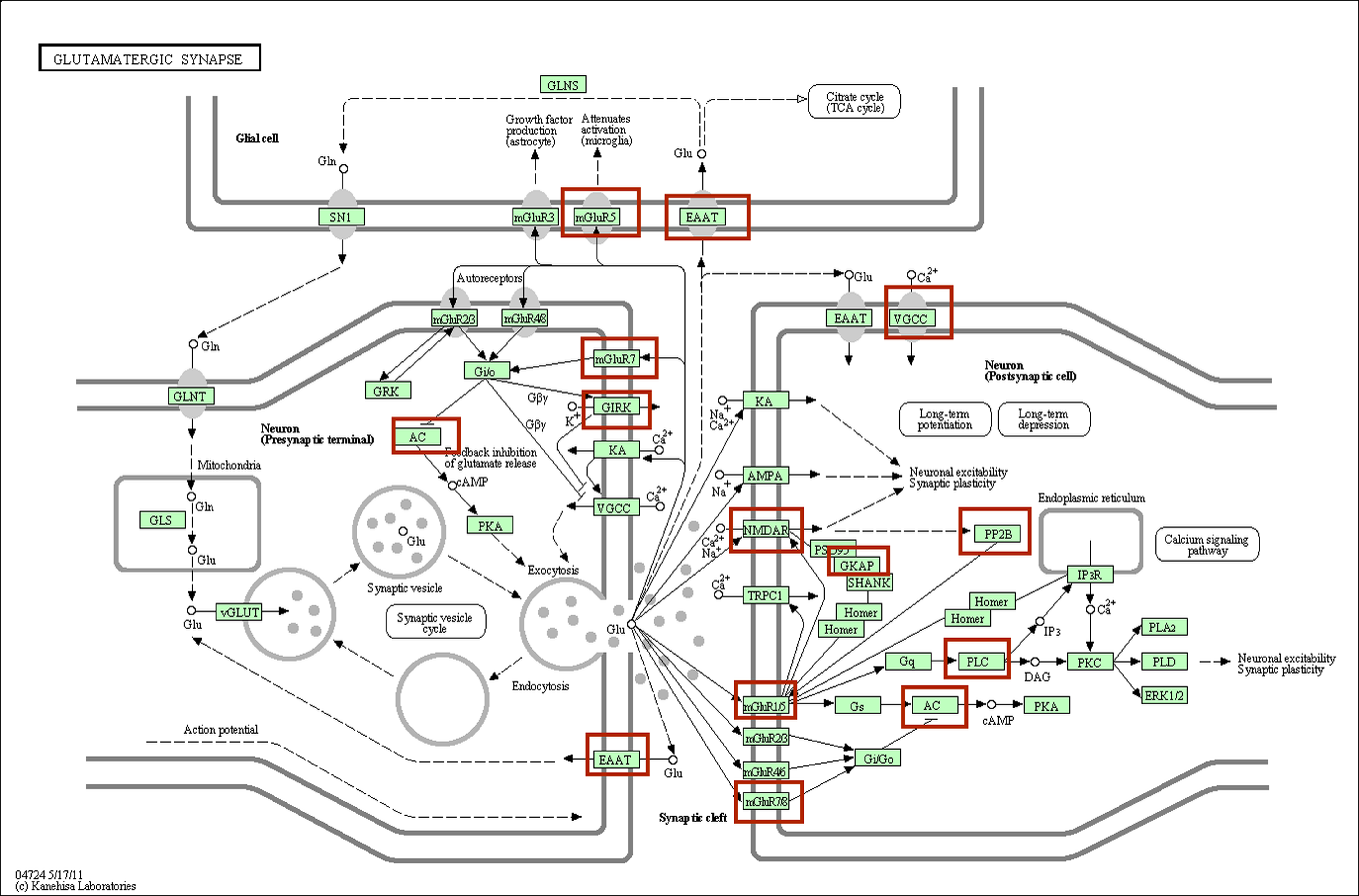

Supplement: S4 Fig — The hsa-miR-137-3p targets predicted by DIANA miRPath v.2.0 in Glutamatergic synapse pathway were shown in red square. NMDA receptor GRIN2A was identifies as potential target. (TIF) [file pone.0137432.s004.tif]

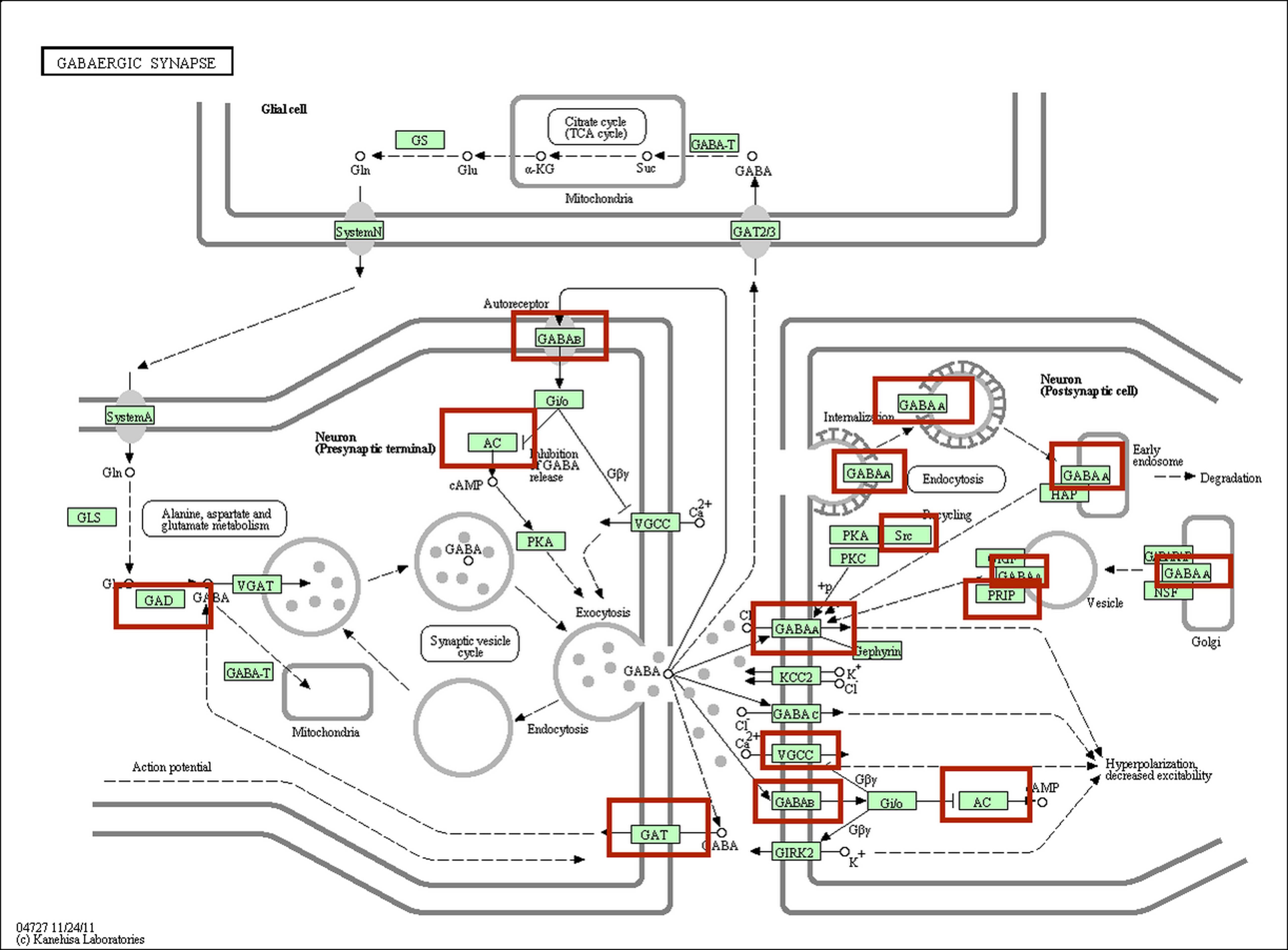

Supplement: S5 Fig — The hsa-miR-137-3p targets predicted by DIANA miRPath v.2.0 in GABAergic synapse pathway were shown in red square. GABA receptors including GABRA1, GABRA6 and GABBR2 were identifies as potential targets. (TIF) [file pone.0137432.s005.tif]
